# A Study on the Application of BP Neural Network Based on Visual Recognition in Regional Economic Forecasting

**DOI:** 10.1155/2022/3531011

**Published:** 2022-09-26

**Authors:** LingYan Meng

**Affiliations:** Universiti Pendidikan Sultan Idris (UPSI), Faculty of Management and Economics, Tanjong Malim 35900, Perak Darul Ridzuan, Malaysia

## Abstract

The economic growth in the new normal is no longer limited to the total amount and scale of economic growth in the traditional and neoclassical periods, but has changed to “quality” and “development” under the dual requirements of historical changes and tasks of the times. The quality of regional economic growth is an important part of the quality of China's economic development and an important part of the quality of China's economic development in the new era. Therefore, this paper proposes a BP neural network based on visual recognition in a regional economic prediction model and conducts application experiments. This regional economic forecasting model is relying on data technology for economic panel data mining, then graphical processing of panel data, followed by the selection of visual recognition technology for economic panel map analysis, to derive its various component coefficients, and finally then using the BP neural network to fit the prediction, at the same time, through long-term and short-term prediction, to predict the future development quality of each region's change trends and fluctuations, to predict the institution's role, so as to avoid major transitions and deteriorating alarms, and to provide support for the macroregulation of regional economic development quality.

## 1. Introduction

For China's economy, 2018 is the beginning of an epoch and a new century and also the “first year of quality,” the quality of economic growth will be anew historical coordinate of China's economic development [1, 2] and also an important milestone of China's economic development, and also the Chinese economy has entered a new period of “quality development” [3]. Looking back, China's economic development has been rapid since the reform and opening up, with the nominal GDP growing at 14.53% per year from 1978 to 2016, with an annual incremental growth rate of 1/3, creating a “Chinese miracle” in the past three decades and laying the foundation for China's Chinese dream. In the twenty-first century, with the restructuring of the global division of labor system, China's strategic position has become increasingly prominent, and China's economic development path is at a new stage of development [4–8].

Looking at the evolution of China's economic development, since the reform and opening up, China has entered a phase of rapid development. During the phase of quantitative growth, China is used to taking the route of “path dependence,” focusing on the results of economic development, under the concept of “cost-benefit,” and putting. The problem of “economic growth” is simply reduced to the problem of “conditions” and “results.” Considering economic growth from the perspective of dynamic evolution, the traditional model of investment accumulation only hopes to obtain continuous income growth, but ignores the multidimensional endogenous scale-reward superposition effect, which is a nonsustainable and uneconomic behavior [9]. Moreover, the early blanket development model, which applied the same growth factor accumulation mechanism to different regions, ignored the fact that the initial resource allocation would have an increasing degree of influence on economic growth as it was gradually driven, leading to large growth effects under the same model. Fortunately, at the end of the twentieth century, the world was thinking about the eternal topic of economic development, and instead of focusing only on the conditions and consequences of economic development, economists tried to give more connotations to economic growth from a larger perspective and proposed an extended concept of economic growth quality to explain the function of endogenous growth paths of economic growth and the accompanying social progress, coordinated development [10], environmental costs, and other deep developmental impacts are comprehensively assessed. We can see that the quality of economic growth is a new development goal, which is a new and contemporary practice of economic growth.

Under the new economic development model, the goal of development has changed from “quantity” to “quality,” which is the need of the times. The report of the 19th National Congress mentions “quality” several times, summarizing China's achievements in recent years while clearly indicating that the main contradiction in China is imbalance and insufficiency. The fundamental contradiction is reflected in the direction of economic development [11]. The goal of economic development has shifted from a quantitative stage of solving the basic food and clothing problem of the population to a higher, green, coordinated, higher and higher level of development, which is an important issue for China's economic development.

Therefore, based on an empirical study on the quality of China's regional economic development, a new development model is proposed, and then the problems of the development path of the quality of China's economic growth at this stage are evaluated, and a feasible way for its future development quality is provided for the future development direction [12].

## 2. Introduction to Related Theories

### 2.1. BP Neural Network Theory

**Figure 1:**
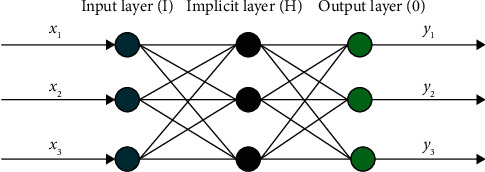
Principle diagram of BP neural network.

BP neural network is one of the most commonly used models, and its use is very wide, but its practical application is mostly only applicable to specific environments compared with our proposed time series model. The BP neural network is based on the intelligence of the human brain, and although it is not fully mature [13], its potential is huge, and it is foreseeable that the human brain is not just a special case in processing information. Therefore, starting from a generalized neural network model, which aims to be applied to the full range of data sequences, it is reasonable to assume that the method can perform good simulations and predictions in many aspects. The BP neural network first consists of three levels of input-output and hidden, while the hidden layer is likely to include multiple levels as well, and multiple nodes can exist on all three levels, and the propagation direction of messages is unidirectional. They are connected to each other by weights. The schematic diagram is shown in Figure 1.

The training of the BP neural network is mainly to process the information of the input layer over the excitation function and get the required information and transform it into the required information and pass it from one layer to another by the excitation function, if there is only one layer, then by the double processing of function and connection weights, that is to get our required result. Then, we decide whether to repeat the intermediate process by comparing our computed output with the real output. Obviously, we can change the excitation function and the number of implicit layers to achieve control over the above process [14].

### 2.2. Visual Identity Theory

Visual identity (VI), also called VIS, is the abbreviation of the English Visual Identity System. Its meaning is to unify and standardize all the visualized corporate images and to communicate the company's image to the public through VI. VI includes corporate name, brand logo, standard fonts, printing fonts, standard graphics, standard colors, slogans, business reports, product descriptions, and software systems. It contains at least ten aspects, i.e., product packaging, production environment and equipment, exhibition space and equipment, means of transportation, office equipment and supplies, work clothes and accessories, advertising facilities and audio-visual products, public relations and gifts, factory flags and signs, instructional signs and signage [15]. Intuitive, communicative, and infectious are the important features of visual identification. Visual identity is to take the basic elements of corporate identity and show them effectively through strong policies and management systems, so as to form the inner visual imagery of the company and convey the spirit and business concept of the company through the unification of visual symbols, thus effectively enhancing the brand image of the company and products.

### 2.3. Data Mining Theory

It is the basic theory of data mining, mining algorithm, data warehouse, visualization technology, knowledge representation, knowledge maintenance and reuse, heterogeneous data mining, web data mining, etc.

The main role of data mining is to discover the types of patterns from data, to predict future trends and behaviors, and thus to provide knowledge-based forward-looking decisions. At present, the main functions of data mining are as follows:

#### 2.3.1. Conceptual Description

It describes the connotation of a specific object and summarizes its relevant characteristics. Specifically, it includes characteristic description and differentiation description; the former refers to the commonality of objects, and the latter refers to the difference between different categories of objects.

#### 2.3.2. Contact Information

Data correlation is a kind of database knowledge with important significance. If two or more variables have a certain pattern, they are called correlations. Association can be classified as simple association, temporal association, and causal association. Correlation analysis aims to discover the network of relationships hidden in the database [16]. The rules generated with association analysis have a certain degree of reliability because the relevant functions of the data in the database are not known. Association analysis finds an association rule that shows the frequent simultaneous occurrence of attribute values in a particular set of data.

#### 2.3.3. Classification and Forecasting

*(1) Category*. It includes categorization and determination of the category based on the properties of the object under test. It is a kind of knowledge that has the characteristics of the same nature and a kind of knowledge that distinguishes the characteristics from other things. Based on this, a classification algorithm based on decision trees is proposed. The algorithm is a decision tree structure based on a collection of examples, which is instructive. There are also statistics, rough sets, neural networks, support vector machines, etc.

*(2) Prediction*. Time series type information is used as the basis for predicting the attribute values or values of a particular sample, based on historical information and new information as input. Traditional statistical methods, neural networks, machine learning, etc., are time series prediction methods.

#### 2.3.4. Cluster Analysis

Cluster analysis is the objective grouping of objects with similar properties according to their attributes. Compared with classification methods, clustering is grouping according to the properties of the data itself, and grouping is carried out without artificial grouping. By clustering or grouping them according to the principles of similarity within majorizing classes and similarity between minorizing classes, the resulting clustering results in greater similarity in the same class and lower similarity between classes.

### 2.4. Theories Related to Regional Economic Growth

Regional economic growth, in a narrow sense, is expressed in monetary terms, that is, the growth of GDP. In a broad sense, regional economic growth also includes controlling population and expanding demand for goods. At present, there are two different development models of regional economic growth in China.

#### 2.4.1. Balanced Development Theory

In the book “Industrialization in Eastern and Southern European Countries,” the British economist Rosensteordan put forward the “Big Push” theory. This theory is a strategic concept for developing countries to start industrialization [17]. It has four main elements. First, at a minimum critical investment scale, synergistic investment in several industrial sectors can achieve “externalities.” Second, investing in complementary industrial sectors to increase industrial output can overcome the problem of narrow markets and shortage of demand in developing countries; simultaneous investment in complementary industries can reduce production costs, improve supply, and promote economic development. Third, vigorously promote domestic and foreign investment from financial sources. Fourth, capital should be invested in capital construction and light industry.

Naxos' strange circle of poverty doctrine. The shortage of capital is an important constraint to the economic growth of developing countries, which is caused by the contradiction between the supply and demand of capital. In the problem of insufficient supply of capital, low income causes low saving capacity, resulting in shortage of capital, which increases productivity and reduces income. From the perspective of capital demand, low income leads to a decrease in purchasing power, which leads to a shortage of investment, the accumulation of capital becomes difficult, and a decrease in productivity, which eventually leads to low income. To break the circle of poverty, it is necessary to increase savings, increase investment, and form reciprocal demand among industries to transform this vicious circle into a virtuous one.

#### 2.4.2. Theory of Unbalanced Economic Development

In his book “A Brief Discussion of the Concept of Growth Poles,” François Perroux puts forward a concept of growth poles based on the imbalance of economic development. The core content of the growth pole theory is that in a certain region, there is a certain innovation capacity, or an important industry, which, to a certain extent, can concentrate capital and technology, so as to achieve a certain scale effect and thus drive the economic development of the surrounding areas. It can be seen that the economic development in China is not balanced and has a polarizing effect.

The growth poles play the role of absorption and diffusion in regional economic development, which is reflected in four aspects, namely, innovation and demonstration, scale effect, external economy, and aggregation [18].

Therefore, for a region to achieve economic development, it is necessary to promote the economic development of its surrounding areas by developing a growth pole and using its polarization and radiation diffusion effects.

The American economist Hirschman, in his Economic Development Strategies, regards the imbalance strategy as the best way to achieve economic development. He argues that economic development is not achieved in all areas, and whenever it occurs in a certain region, it concentrates economic growth in the original direction of development. The formation of such growth poles inevitably leads to unevenness between regions, and in areas where regional power is concentrated, priority tends to be given to development.

This theory provides the theoretical basis for our economic development, development mechanism, and government intervention. Based on the realistic economic growth and development mechanism, it provides new ideas for the economic development of less-developed regions. However, it ignores the lack of markets and social, economic, and infrastructure development in less developed regions, which leads to the ineffective operation of various incentive mechanisms.

## 3. Application Method Design

### 3.1. Regional Economic Data Mining Model

**Figure 2:**
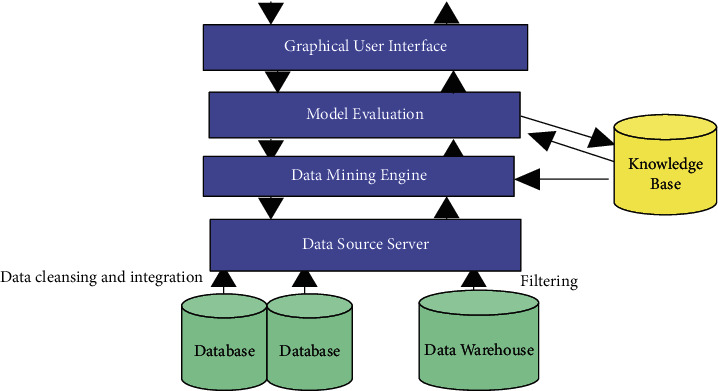
Data mining model.

The research on economic forecasting from the regional perspective of China's macroeconomic forecasting mainly focuses on the construction of the regional economic forecasting system. However, before regional economic forecasting, data need to be obtained and processed first. The model diagram of this data mining system is shown in Figure 2.

#### 3.1.1. Data Source

A data source provides data needed for data mining, including a database or set of databases, data warehouses, spreadsheets, and other types of information repositories.

#### 3.1.2. Data Source Server

It filters, cleans, and integrates data sources to meet users' needs for data mining.

#### 3.1.3. Data Mining Engine

A data mining engine is the core part of the system, and it includes several functional modules such as characterization, association, classification, cluster analysis, evolution, and deviation analysis.

#### 3.1.4. Pattern Evaluation

Eliminating unnecessary or redundant patterns and filtering them based on thresholds of interest to obtain interesting patterns. GUI: an interactive interface between the system and the user that presents interesting patterns (knowledge) in various forms in an intuitive way, such as charts and tables.

#### 3.1.5. Knowledge Base

The knowledge base is stored, and guides the process of data mining and the interpretation and evaluation of models.

### 3.2. Regional Economic Forecasting Model Construction

Since regional economic development is nonlinear, unstable, and unbalanced, it is difficult to ensure its effectiveness with linear methods.

From a long-term perspective, the system established should not only be in line with the comparative level of the eight balanced regions but also with the overall development trend during the new normal.

In the short term, the forecast of the quality of regional economic development should aim to meet the needs of time and individuals. First, monthly data should be studied in order to achieve the timeliness of regional economic development quality forecasting. Secondly, the purpose of short-term forecasting is different from the long-term preference trend, where the study from the individual perspective focuses on the fluctuations of individual regions and requires the establishment of a unified and uniform logical system under the main forecasting indicators [19]. Under the requirement of these two objectives, this paper proposes a coarse-set-based preprocessing method by simplifying the coarse set and synthesizing it into a comprehensive forecasting index, which can reduce the complexity of the information system and optimize the subsequent system effectively while ensuring the same forecasting performance.

**Figure 3:**
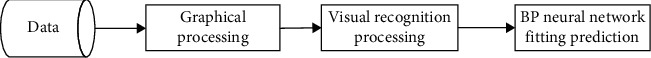
Diagram of the economic forecasting model.

This regional economic forecasting model is based on data technology for economic panel data mining, then graphical processing of the panel data, followed by the selection of visual recognition technology for the analysis of the economic panel map to derive its component coefficients, and through the synthesis of the four main aspects of the index, a BP neural network is used for forecasting. Its model diagram is shown in Figure 3.

## 4. Application of Experimental Analysis

### 4.1. Data Preparation

The experimental data in this paper are all from China Economic Network, based on the data from 2000 to 2021; the most important data are the important factors affecting regional GDP growth. Using data mining methods, from monetary policy, investment and consumption, exchange rate, total retail sales of social goods, urbanization process, science and technology innovation, etc., a total of 963,025 pieces of data were mined, and finally, by using data mining and data processing in the model, the data according to regions and forecast indicators were classified [20].

### 4.2. Regional Economic Fitting and Forecasting Experiment

The regional economic fitting prediction experiment is a fitting prediction model based on the visual recognition technology with eight nodes as the input layer, and the results are shown in Table 1.

It can be seen that for the selection of eight input layer nodes, the best result of 20-40-20 is 20-40-20. This indicates that the internal structure mechanism of the neural network of 20-40-20 can better explain the inner rules of this time series.

**Figure 4:**
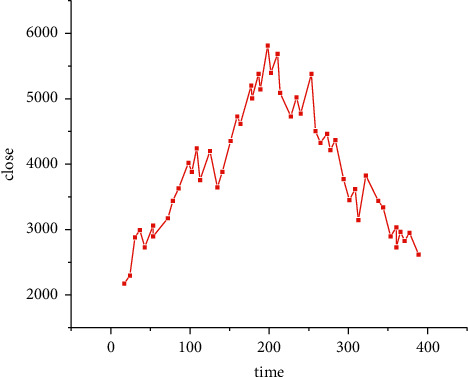
Fitted prediction diagram for the eight nodes of the input layer.

Figure 4 shows the prediction fit image of this time series with eight nodes in the input layer and 20-40-20 in the implied layer (the red line is the fitted curve, and the black line is the real curve). As we can see, the prediction results are excellent.

### 4.3. Short-Term Trend Forecasting of China's Regional Economy

**Figure 5:**
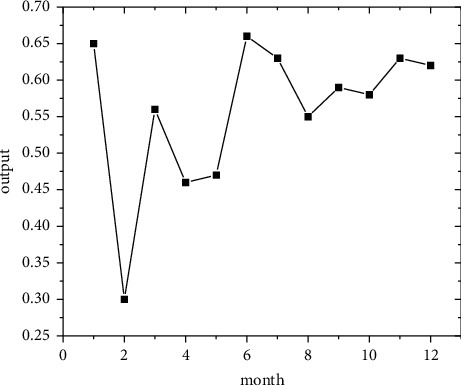
North Coast monthly economic growth quality forecast, 2018.

**Figure 6:**
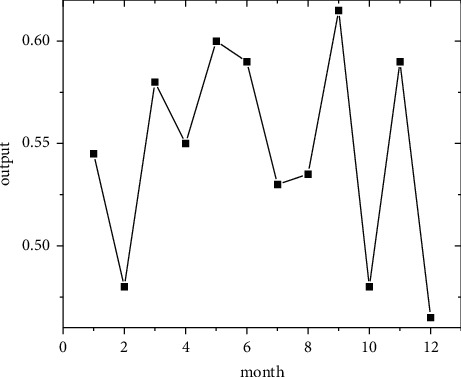
Eastern Coast monthly economic growth quality forecast, 2018.

**Figure 7:**
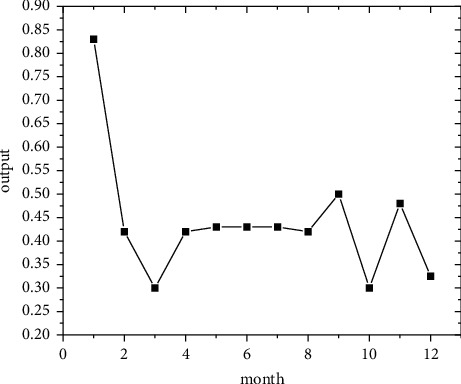
South Coast monthly economic growth quality forecast, 2020.

**Figure 8:**
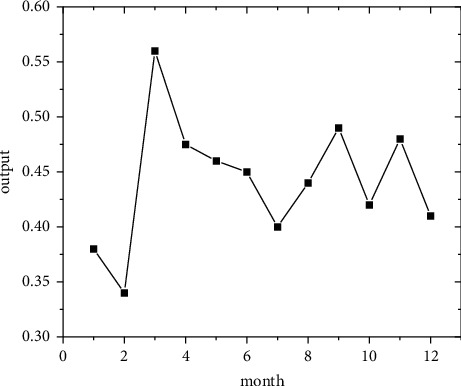
Northeast monitoring monthly economic growth quality forecast, 2020.

**Figure 9:**
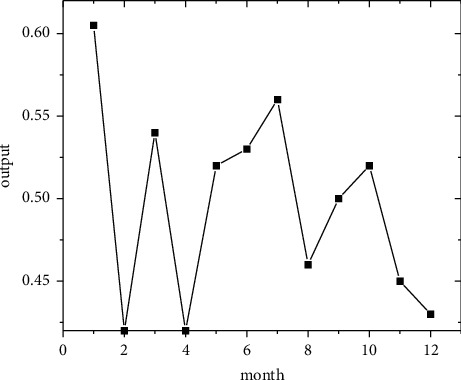
Monthly economic growth quality forecast for the Central Yellow River in 2020.

**Figure 10:**
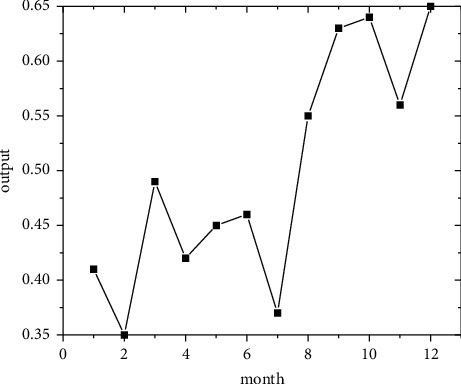
Central Yangtze River monthly economic growth quality forecast, 2020.

**Figure 11:**
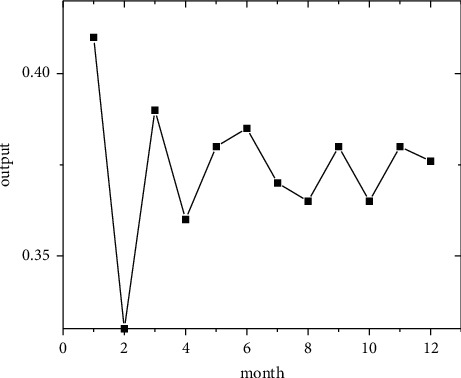
Southwest monthly economic growth quality forecast, 2020.

**Figure 12:**
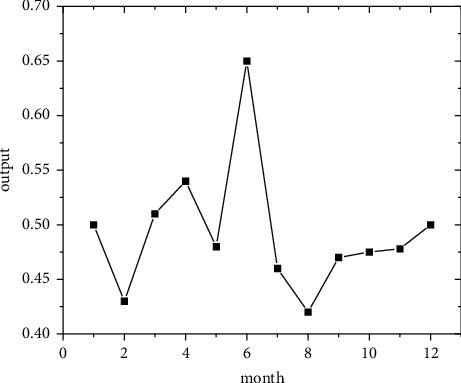
Great Northwest monthly economic growth quality forecast, 2020.

In the short term, forecasts are made using the regional economic forecast model, and by modifying the monthly information from 2011 to 2019, the best eight regions are selected among the eight regions for optimal forecasting [21]. Output the forecast values of the quality of economic growth of major regions from January to December 2020, as shown in Figures 5–12.

Looking at the short-term forecast, there is a clear downward trend in all regions around the Chinese New Year in February this year, which is in line with the basic pattern of the economy. There is a slight increase in volatility throughout the year. According to this forecast, in addition to the traditional first-quarter volatility, the North Coast will also see a brief downturn in the second quarter, followed by an upward phase of volatility [22]. Similar to 2019, the eastern coast has 3–4 phases of large waves with a major trough in October. The southern coast is generally showing a high start to 2020, with insufficient momentum for subsequent improvement, and has not yet recovered to its starting level. The Northeast started the year or was affected by last year's volatility, with a low starting point for economic growth and a sawtooth shape in the third and fourth quarters, with an unfavorable final fall. Compared with 2017, the economic development of the Central Yellow River region is noteworthy, with two serious recessions in February and March, and in the subsequent adjustment, the momentum effect is not obvious, and the fall point at the end of the year is low, which may have an impact on the beginning of next year. The Central Yangtze River region has outperformed this year, and after the volatility of the first two quarters, the third and fourth quarters are likely to be much better. The monthly trend in the Southwest region is similar to 2019, with a relatively small range in all months except the first quarter, with steady economic growth, but the overall pull-up mechanism is weak in its role and needs to be consistently improved. This year, June is the peak of the great northwest region, excluding the anomaly, there is a certain pulling effect around the middle and third quarter of each year, presumably the second and third quarter intersection for the region to enhance the quality of economic growth opportunity, and the subsequent trend has far-reaching impact. Future regulation can be advanced in this point in time, in order to make time judgments from the forecast perspective, reversing the long-term trend.

### 4.4. Long-Term Trend Forecast of China's Regional Economy

In the long-term forecast, we focus on the overall development trend of regional economic development, try to dig out the rate of improvement or slowdown from the global perspective, and analyze in detail the differentiation status among individual regions and the possibility of achieving regional catch-up[23]. Meanwhile, using the established regional economic forecasting model, the regional economic development quality forecasting indexes from 2000 to 2021 are divided into five stages for cyclic learning and correction within five years, and finally, the implied level is set as a single level with the number of neurons as 5, so as to obtain the development trend of five years.

**Figure 13:**
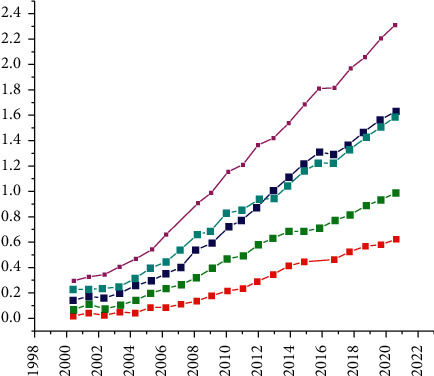
Long-term trend projections for the quality of economic growth in the eight regions.

The black part in Figure 13 shows the real quality in the past, and the gray one is the expectation for the future. From the time dimension, we can see that the quality of economic development in the eight regions will continue to improve in the longer term. According to the annual historical analysis of the three ladder pairs, the North and East coasts remain at the top of the rankings, and since 2017, the quality of economic growth in these two regions will level off, while the East Coasts will continue to take advantage of efficiency gains and have better overall quality than the North Coasts. There will be changes in the configuration of the second tier. In the past 17 years of quality forecasts, the southwest region has continued to maintain its growth potential and will surpass the middle reaches of the Yangtze River in the future, officially ranking among the second tier. The original South Coast and the two midstream regions will see little improvement in the quality of economic growth over the next five years, proving that there is spatial overlap between the three regions mentioned above. In the early stage of economic development, the south coast police situation is dominant and weak in terms of operational processes, results, and benefits, and its priority is the efficiency-driven quality improvement path, and its future quality status is still ahead of the two midstream regions, but it is also necessary to pay attention to the insufficient growth base caused by the long-term revaluation state of the quantity and quality of input factors, and the level of human capital and physical capital supply after the efficiency improvement of industrial upgrading The match with the level of human capital and physical capital supply after the efficiency of industrial upgrading, with a view to continue to maintain consistency with the level of quality of the first tier. In the two mid-tier regions, the tendency is to adopt the path of improving quality in the investment phase, which is effective but does not generate endogenous economic growth momentum in the long run, and when conditions accumulate to a certain extent, a distortion, slow and digestive capacity distortion, will be generated, and it will take more time and effort to improve production efficiency by then. The quality of the third echelon in the Northeast is still slow to improve, and the problem of transformation and upgrading will continue to be a structural resistance; in the short term, the success of the Great Northwest to solve the problem of locking the lower end of the quality level also depends on the improvement measures in many aspects of development conditions, processes, results, and benefits, and at the level of strategic adjustment.

Overall, the first tier of regional quality remains stable and continues to lead over the next five years. The second tier, with the addition of Southwest and the stability of the three regions, is expanding significantly, but is encountering many obstacles to break through to the first tier. The third level continues to improve but lacks effective incentives and innovative ways to achieve great growth, and the superior engine for achieving great growth has not yet taken shape, and the path to improvement remains elusive. Based on the forecast and analysis of the history and current situation of economic development, how to break the status quo, stabilize the advantages, tap new effective growth engines, and achieve a dynamic balance of high quality is the key to quality regulation in the future.

### 4.5. Policy Shift of China's Regional Economic Growth Quality Improvement

The transformation of regional economy from quantitative growth to qualitative growth requires mutual coordination between national macrocontrol and regional macrocontrol. At the level of conditions, we should actively explore the new structural characteristics of factor endowment, comply with the background of the new normal economic development law, and improve the quality of supply; take health-oriented financial capital, and guide the flow of capital to the real and emerging green industries by reducing the financial leverage of each region; and take a flexible approach directing human capital flows to high-tech and innovative industries. At the process level, reforms are used to further develop market dynamics, tap potential growth drivers in the transition period, and effectively innovate the social security system to maintain a smooth, fair, stable, and green operating mechanism and an environment for efficiency. To measure the value judgment of regional economy and development from the perspective of evolutionary results and radiating benefits, to bring into play regional synergy under the logic of development and quality, and to achieve dynamic balance in the per capita sense under the guidance of national macropolicies, complemented by differentiated macrocontrol policies with long and short-term interaction matching the regional quality status.

To achieve all-round quality control in the whole process of economic development, the system of rapid development such as total control, individual regulation, speed regulation, and short-term regulation must be abandoned. On the basis of the new normal and the characteristics of the new era, according to the characteristics of the eight regions, the focus of regional development is on a unified logic, long-term and short-term interactive regulation, the regulation of coordinated development, and effective low-end supply improvement. We should put the regional characteristics of unified and differentiated regulation ideas throughout each regulation and steering measures, accelerate the establishment of regional economic, scientific and technological innovation, and financial and human resources coordinated the development of the regional industrial system, and build a regional economic market mechanism effective. Regional micro subjects have vitality, regional macro regulation, and control of the economic system, and constantly enhance the innovation and competitiveness of China's regional economy, to achieve long-term sustainable regional economic growth. The regional economic innovation and competitiveness of China's regional economy will be continuously enhanced, and long-term sustainable regional economic growth and all-round quality improvement will be achieved.

## 5. Conclusion

The transformation of regional economy from quantitative growth to qualitative growth requires mutual coordination between national macrocontrol and regional macrocontrol. At the condition level, we should actively explore the new structural characteristics of factor endowment, comply with the background of the new normal economic development law, and improve the quality of supply; health-oriented financial capital by reducing the financial leverage of each region, guide the flow of capital to the real and emerging green industries; and guide the flow of human capital to high-tech and innovative industries in a flexible manner. Economic forecasting is the application of the theory and method of forecasting to economic activities and the study of future economic phenomena. This paper uses theories and methods from several disciplines, including economics, probability theory, mathematical statistics, modern management science, system theory, and computer science, and applies appropriate mathematical modeling techniques to analyze and forecast the development trend of the research object.

This paper starts from the basic theoretical framework of macroeconomic growth quality, summarizes the current research basis and shortcomings from the empirical point of view, reconstructs the research idea of regional economic growth quality, therefore proposes the BP neural network in the regional economic forecasting model in visual recognition, and uses data mining technology for data preparation and the regional economic data set for model fitting. The model is then fitted to the regional economic dataset for prediction experiments and long-term and short-term economic prediction experiments, and finally, it is concluded that the model is well applied and has good feedback for long-term and short-term regional economic prediction.

## Figures and Tables

**Table 1:** Fitted prediction test table.

Number	Network structure	Decidability factor			
		Training set	Validation set	Test set	Total data set
1	2	0.8276	0.8504	0.8425	0.8373
2	5	0.8125	0.7713	0.8508	0.8082
3	5–8	0.8454	0.7614	0,8617	0.8365
4	5–10	0.8321	0.8764	0.8443	0.8245
5	10–20	0.8354	0.9026	0.7714	0.8321
6	40–40	0.8754	0.8675	0.8865	0.8767
7	50–100	0.8435	0.9123	0.8454	0.8367
8	100–200	0.8504	0.8243	0.8941	0.8588
9	200–400	0.8478	0.8759	0.8132	0.8587
10	20-4020	0.9443	0.9156	0.9633	0.9456
11	50-100-50	0.8697	0.8367	0.8388	0.8553
12	20-40-40-20	0.8723	0.8505	0.8061	0.8343
13	10-20-20-10	0.8542	0.9578	0.8937	0.9032
14	10-20-20-20-10	0.8521	0.9352	0.9389	0.9405

## Data Availability

The dataset used in this paper are available from the corresponding author upon request.
